# Amorphous Solid Dispersions in Non-Oral Drug Delivery: A Critical Review Bridging Mechanistic Advantages and Gaps in Translational Evidence

**DOI:** 10.1007/s11095-026-04200-6

**Published:** 2026-09-17

**Authors:** Bruno Vincenzo Fiod Riccio, Aline Franciane Leão, Ana Beatriz Klosowski, Fernanda Isadora Boni, Leonardo Miziara Barboza Ferreira, Maurício Palmeira Chaves de Souza, Priscileila Colerato Ferrari, Marlus Chorilli

**Affiliations:** 1 School of Pharmaceutical Sciences, Department of Drugs and Medicines, São Paulo State University (UNESP), Araraquara-Jaú Hwy., , Km 1, Araraquara, São Paulo, 14800-901 Brazil; 2 Department of Pharmaceutical Sciences, Ponta Grossa State University (UEPG), General Carneiro Ave., , Ponta Grossa, Paraná, 4748, 840230-900 Brazil; 3 School of Pharmaceutical Sciences, University of São Paulo (USP), Av. Professor Lineu Prestes, São Paulo, SP 05508-000 Brazil

**Keywords:** mechanistic drivers, molecular interactions, regulatory framework, supersaturation, translational constraints

## Abstract

**Background:**

Non-oral drug delivery routes, including cutaneous, transdermal, pulmonary, ophthalmic, parenteral, vaginal, and rectal administration, are essential for both local and systemic therapies but impose route-specific constraints on drug dissolution, retention, permeation, clearance, and exposure. Amorphous solid dispersions (ASDs),extensively investigated for oral delivery, may improve the performance of poorly water-soluble drugs by stabilizing high-energy amorphous states, increasing apparent solubility, and promoting transient supersaturation. However, their translation into non-oral dosage forms remains fragmented and largely preclinical.

**Objective:**

This review critically examines the potential, limitations, and translational challenges of ASDs for non-oral drug delivery, with emphasis on the physicochemical, biopharmaceutical, pharmaceutical, and route-dependent determinants governing their performance.

**Scope:**

Key aspects discussed include drug–polymer interactions, molecular mobility, phase behavior, water uptake, crystallization, interactions with secondary pharmaceutical bases, and route-specific pharmacokinetic/pharmacodynamic relationships. Current evidence is examined across dermal, transdermal, pulmonary, ophthalmic, vaginal, rectal, and parenteral delivery systems. Parenteral administration is considered a special case encompassing sterile suspensions, reconstitutable systems, microneedles, implants, and depot-forming formulations. Recent technological advances, patent and industrial landscapes, and the potential extension of ASD principles to peptides, proteins, biologics, and other complex molecular entities are also addressed. ASDs are further positioned in relation to alternative and complementary solubility-enhancing technologies, including nanoparticles, nanocrystals, cocrystals, cyclodextrins, micelles, lipid-based systems, emulsions, solvents, and cosolvents.

**Conclusions:**

ASDs off er a promising but still underdeveloped strategy for improving non-oral delivery of poorly soluble drugs. Their successful translation will depend not only on achieving and maintaining favorable amorphousstates and supersaturation, but also on converting these physicochemical advantages into route-specific drug exposure, therapeutic benefit, formulation stability, manufacturability, and clinically meaningful outcomes.

**Graphical Abstract:**

**Figure:**
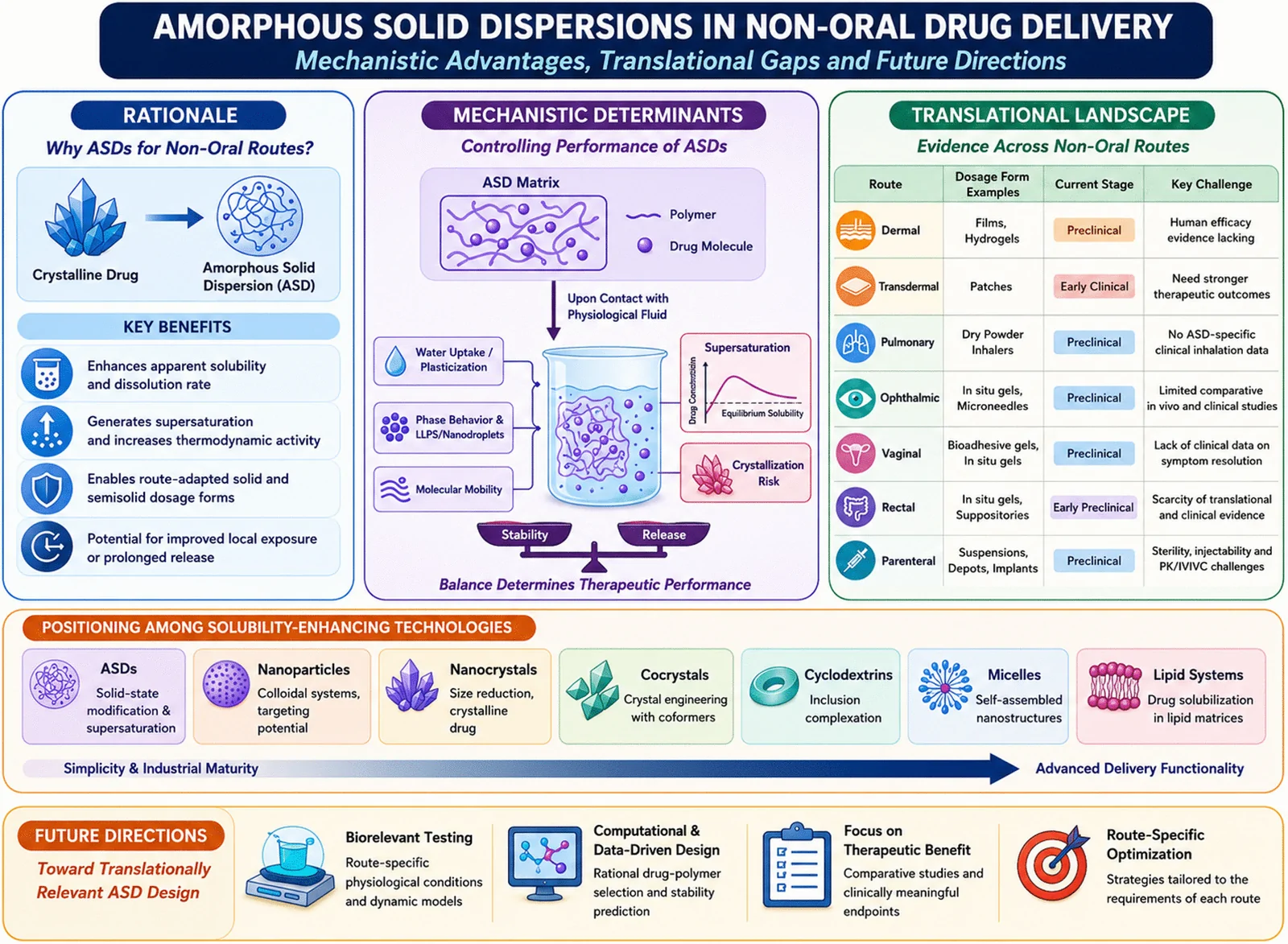

## Introduction

Non-oral drug delivery routes, such as cutaneous, transdermal, pulmonary, ophthalmic, parenteral, vaginal, and rectal administration, are vital in modern medicine. They allow for localized treatment, minimize systemic side effects, and can enhance patient adherence. Nonetheless, these routes are not uniform within the biopharmaceutical domain [1].

Each epithelial interface has unique anatomical and physiological features that influence drug dissolution, retention, permeation, and clearance. For example, the *stratum corneum* limits skin penetration, tear turnover affects eye retention, and pulmonary delivery depends on particle deposition and dissolution in alveolar lining fluid. These barriers demonstrate that drug efficacy in non-oral routes depends on specific microenvironmental conditions rather than solely on drug properties [2–4].

The challenge is especially significant for poorly water-soluble compounds, which make up a large share of new chemical entities. Therapeutic failure often results from inadequate local drug exposure rather than insufficient pharmacological potency [5].

Factors such as limited fluid volume, rapid clearance, and restricted permeability can hinder complete dissolution and diminish the free-drug concentration, that is, the thermodynamic driving force for tissue permeation. Therefore, formulation strategies must focus not only on improving solubility but also on managing the complex interactions between dissolution kinetics, local concentration gradients, and biological barriers to ensure effective therapeutic results [6].

Amorphous solid dispersions (ASDs) have emerged as one of the most effective formulation approaches for improving the performance of poorly soluble drugs, particularly in oral delivery, which is far better studied than other routes. By stabilizing drugs in a high-energy amorphous state within a polymeric matrix, ASDs can enhance apparent solubility, accelerate dissolution, and promote transient supersaturation [7].

However, their performance is not governed solely by amorphization. Intermolecular interactions between drug and polymer, molecular mobility, and phase behavior critically determine the stability of the amorphous state and the maintenance of supersaturation. These interactions can simultaneously enhance stability and modulate release kinetics, underscoring the need for a balanced design that balances stabilization and drug release [8].

Despite the robust mechanistic understanding of ASDs, their application beyond oral drug delivery remains virtually unexplored, especially from a translational perspective. The success of ASD-based formulations depends on their ability to generate and sustain supersaturation under highly constrained physiological conditions, including limited fluid volumes, rapid clearance, and variable permeability [9].

Moreover, traditional *in vitro* characterization methods often fail to capture these dynamic environments, limiting their predictive value. Emerging translational approaches emphasize integrating biorelevant *in vitro* models, mechanistic understanding, and in silico tools to better anticipate *in vivo* performance [10].

Although the main body of ASD research still focuses on poorly water-soluble small-molecule drugs, emerging technologies and formulations involving peptides, proteins, biologics, and other complex molecular entities. This is also relevant to the future development of non-oral amorphous systems [11].

Taken together, these considerations suggest that ASDs should be reconsidered not merely as solubility-enhancing systems, but as mechanistically driven platforms whose therapeutic success depends on their alignment with route-specific biopharmaceutical constraints. In this context, the key translational challenge is not the generation of supersaturation per se, but the ability to translate this physicochemical advantage into consistent, clinically relevant drug exposure across different non-oral routes.

This review aims to critically examine the mechanistic foundations of ASD performance, analyze their applicability across non-oral delivery systems, compare with other solubilization-enhancing systems, and identify the current gaps in translational evidence that limit their broader clinical adoption. Databases including PubMed, Medline, NIH, Scopus, Science Direct, and Google Scholar were assessed to write this review. The primary descriptors were as follows: “solid dispersion” or “amorphous solid dispersion” in combination with “supramolecular properties”, “translational”, “administration”, and “clinical trial”.

## Concept and Physicochemical Attributes of Non-Oral Amorphous Solid Dispersions

The effectiveness of ASDs depends on several interconnected mechanistic factors that go beyond merely transforming a crystalline drug into an amorphous state. While amorphization increases free energy and can boost apparent solubility, the actual pharmaceutical effectiveness of an ASD hinges on its ability to remain physically stable during storage while still allowing rapid, relevant drug release upon administration [12, 13]. Table 1 summarizes the mechanistic drivers of ASDs.

**Table 1:** Mechanistic drivers governing ASD performance in non-oral drug delivery

Determinant	Mechanistic role	Impact on performance	Trade-off/risk	Relevance for non-oral delivery	References
Drug–polymer interactions	Promote miscibility and stabilize the amorphous state	Delay crystallization and modulate release kinetics	Strong interactions may hinder drug release	Critical under limited fluid conditions	[13, 16, 17]
Phase behavior during hydration	Governs structural evolution upon contact with fluids	Determines dissolution pathway and release consistency	Phase separation may impair performance	Highly relevant in mucosal and low-fluid environments	[12, 13, 15]
Liquid–liquid phase separation (LLPS)	Formation of drug-rich nanodroplets/colloids	May sustain apparent supersaturation	May reduce free-drug fraction or promote instability	Important for interpreting dissolution outcomes	[12, 14, 15]
Supersaturation	Increases chemical potential and driving force for diffusion	Enhances potential permeation and exposure	Metastable; prone to precipitation/crystallization	Central advantage of ASDs	[12, 15]
Congruent vs non-congruent release	Determines synchronization of drug and polymer dissolution	Affects release uniformity and surface dynamics	Non-congruent release may form drug-rich layers	Important for predicting route-specific behavior	[13, 17]
Drug-rich colloids	Act as transient reservoirs during dissolution	May prolong apparent drug availability	May not represent the bioavailable drug fraction	Relevant for biopharmaceutical interpretation	[14, 15]
Molecular mobility	Controls molecular rearrangement and nucleation	Influences crystallization tendency	High mobility promotes instability	Links stability and in-use performance	[18, 20]
Water uptake/plasticization	Lowers Tg and increases molecular mobility	May accelerate release or destabilization	Promotes crystallization under humid conditions	Critical in dynamic physiological environments	[18, 19]
Crystallization kinetics	Defines the rate of amorphous-to-crystalline transition	Impacts shelf-life and performance consistency	Rapid crystallization reduces efficacy	Important when the exposure window is short	[18]–[20]
Stability–release balance	Integrates stabilization and release mechanisms	Determines overall pharmaceutical performance	Excess stabilization may limit release	Core design challenge for ASDs	[12, 13, 17, 20]

ASD: Amorphous solid dispersion; LLPS: Liquid–liquid phase separation; Tg: Glass transition temperature

**Fig. 1:**
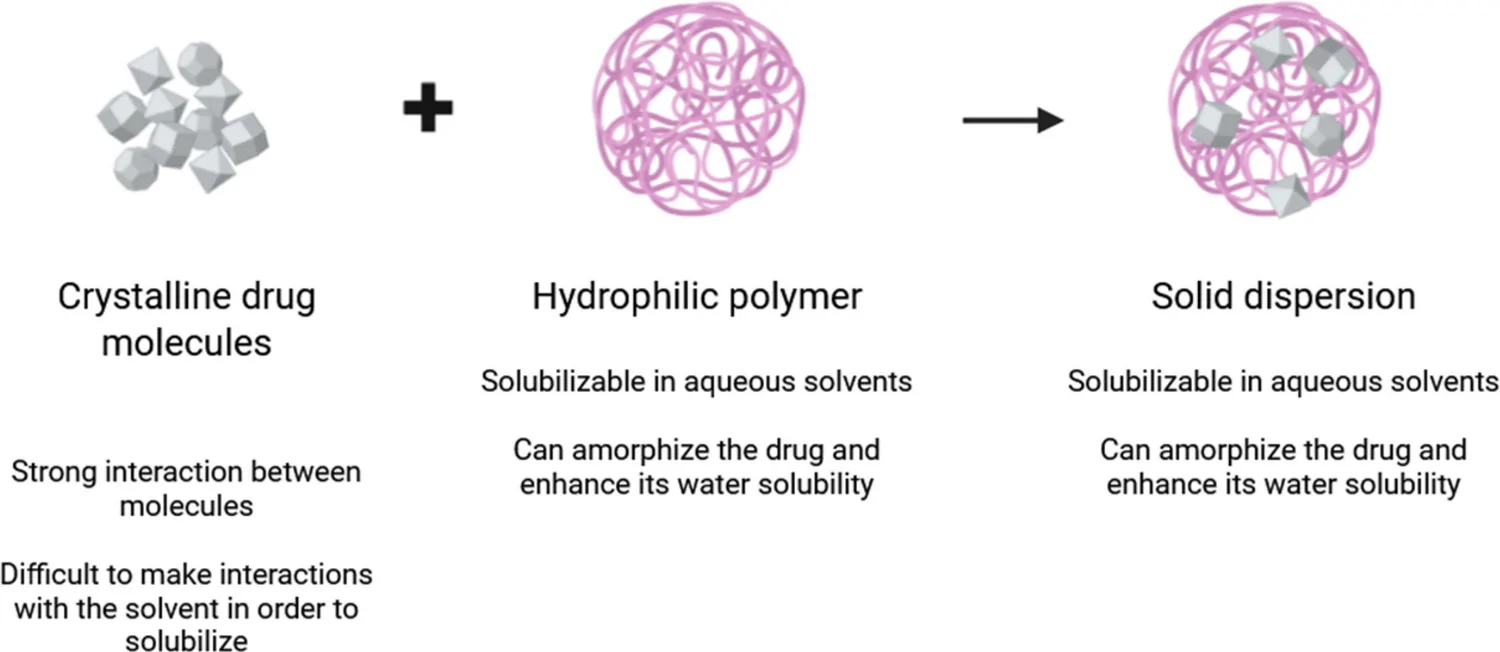
Conceptual representation of solid dispersion systems.

**Fig. 2:**
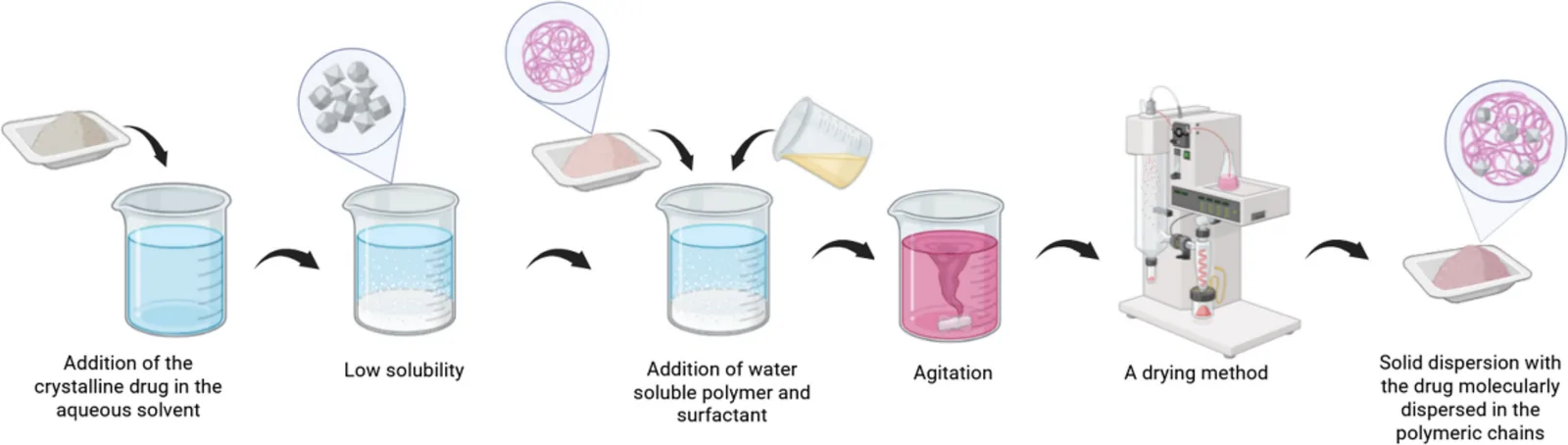
Preparation process of a solid dispersion system.

ASD performance should be viewed as a dynamic interplay involving drug-polymer interactions, molecular mobility, phase behavior during hydration, and dissolution performance relevant to the route. Only by reaching this equilibrium can a formulation avoid phase separation and allow efficient drug delivery [12, 13]. An illustration regarding the concept representation of ASDs is exhibited in Fig. 1, while the preparation process is displayed in Fig. 2.

### Drug–Polymer Interactions and Phase Behavior

Drug–polymer interactions are essential in ASD design, affecting miscibility, stability, and drug release. However, they should not be seen only as static stabilizers in the dry matrix. During hydration, the formulation may change in local composition, water absorption, and molecular organization, thereby modifying the drug-polymer relationship. The quality of an ASD depends not only on initial molecular dispersion but also on how the system changes upon contact with the dissolution medium [13, 14].

This evolving behavior is especially significant because the dissolution of ASDs often involves phase transitions, not just straightforward one-step dissolution. Recent research indicates that supersaturated dissolution can cause liquid–liquid phase separation and the formation of drug-rich nanodroplets or colloidal phases [12, 15].

These entities may serve as temporary reservoirs that maintain the maximum free drug concentration, but they can also lead to further instability, depending on their composition, growth, and interactions with the polymer. Therefore, the phase behavior during dissolution should be viewed as a key mechanistic factor influencing ASD performance, rather than a mere secondary effect [14, 15].

Simultaneously, the ability to rationally choose polymers has become increasingly important. Computational methods, such as COSMO-SAC, for predicting compatibility have shown that polymer selection can be based on thermodynamic compatibility rather than on empirical screening alone. However, compatibility in ASD systems should be approached with caution, as good miscibility in the dry state does not necessarily ensure ideal dissolution upon hydration, which can trigger phase separation [16].

For larger molecular entities like peptides and proteins, drug–polymer interactions can extend beyond mere miscibility and crystallization prevention. They may also involve stabilizing the molecule's conformation, reducing molecular mobility, shielding it from interfacial stress, and preventing aggregation during processing and storage [11].

### Supersaturation, Release Pathways, and Drug-Rich Systems

Supersaturation is one of the main functional advantages of ASDs because it increases the dissolved drug's chemical potential, thereby increasing the driving force for diffusion and permeation. However, supersaturation is inherently metastable. Its contribution to performance depends not only on the amount of drug released, but also on how long the elevated free-drug concentration can be maintained before the system undergoes precipitation, phase separation, or surface-mediated crystallization [12, 15].

Because of this, ASD dissolution should not be viewed as a single, universal process. The release pattern can vary with composition and drug loading, resulting in either more synchronized (congruent) or less synchronized (non-congruent) release rates of the drug and the polymer [13, 17].

Congruent release is generally considered more advantageous because it minimizes the formation of a drug-enriched interfacial layer that can crystallize or block further release. Conversely, non-congruent release may lead to the buildup of drug-rich, less mobile domains near the particle surface, thereby inhibiting further drug release [17].

The role of drug-rich colloids adds complexity to this understanding. In some cases, these colloids maintain supersaturation and enhance drug release; in others, they indicate a failure mode where the formulation cannot supply sufficient molecularly dissolved drugs for effective transport [14, 15].

Consequently, forming nanodroplets or colloids should not be automatically deemed beneficial or problematic. Their significance depends on whether the colloidal phase maintains pharmaceutically active free-drug levels for the necessary duration [14].

### Molecular Mobility, Water Uptake, and Crystallization Kinetics

The physical stability of ASDs is closely related to molecular mobility. Crystallization involves molecular rearrangement, and the extent to which the polymer matrix restricts local and long-range mobility directly influences nucleation and crystal growth. In this sense, the practical effectiveness of an ASD depends not only on its amorphous state at the time of production but also on its ability to resist structural changes during storage and use [18, 19].

Recent research has clarified that crystallization in ASDs depends on both temperature and molecular mobility, though this relationship varies among different polymer types. Time–temperature–transformation studies indicate that each drug–polymer combination has a specific temperature range where crystallization occurs most rapidly, reflecting a balance between molecular mobility and the energy barrier to crystallization [18, 20].

Additionally, humid environments can accelerate destabilization by increasing water absorption, plasticizing the matrix, and promoting both sorption and crystallization. These results highlight that ASD stability is a kinetic issue influenced by environmental and formulation factors, rather than a fixed property of the material itself [18, 19].

From a translational perspective, this issue is quite significant because shifts in mobility and crystallization tendency can impact shelf life and drug release profiles during use. In non-oral applications, where the ASD is usually incorporated into drug formulations, fluid exposure is limited, and interaction times are short, even small changes in crystallization rates can lead to notable differences in local drug availability. Thus, molecular mobility acts as a mechanistic link between solid-state stability and therapeutic consistency [18, 20].

### The Mechanistic Balance Between Stability and Release

A common challenge in ASD development is that factors enhancing solid-state stability do not always improve drug release. Stronger drug–polymer interactions, decreased mobility, and minimized phase separation can prevent crystallization, yet these same traits might also impede matrix hydration, slow down polymer dissolution, and limit drug release into the surrounding medium [17, 20].

On the other hand, systems that dissolve quickly and reach high supersaturation are often more susceptible to issues such as interfacial drug enrichment, colloid formation, and crystallization later. In this regard, interfacial drug enrichment and colloid evolution should also be considered during ASD formulation [12, 13].

A successful ASD design should not focus solely on maximum stabilization or maximum release. Instead, it should aim for a mechanistically appropriate balance between these two aspects. This is particularly crucial for non-oral drug delivery, where the ideal release profile depends on route-specific factors such as the usually limited fluid availability, clearance rates, and barrier properties [13, 17].

In this context, the key value of ASDs lies in their flexibility: they are not just amorphous carriers. Rather, they are adaptable systems whose performance results from the coordinated regulation of drug–polymer interactions, phase behavior, molecular mobility, and dissolution pathways [16, 20].

## Biopharmaceutical Determinants of Translational Performance in Non-Oral ASDs

The practical significance of ASDs in non-oral drug delivery depends not only on their ability to achieve supersaturation but also on how well they enhance drug exposure at the administration site under physiological constraints. In this context, biopharmaceutical performance should be viewed as a combination of solubility, free-drug availability, tissue partitioning, local retention, and route-specific clearance [21–24].

Consequently, an ASD that performs well in a standard dissolution test might not offer significant therapeutic benefit if its release profile does not align with the biological time window and barrier characteristics of the target tissue. In this sense, some routes lack tests capable of accurately predicting ASD solubilization and release, as the *in vitro* dissolution test is validated for the oral route [25–27].

### Secondary Pharmaceutical Bases as an Additional Translational Layer

For most administration routes, ASDs are not used alone as pure solid materials. Instead, they are typically integrated into secondary pharmaceutical bases or dosage-form platforms, such as creams, ointments, hydrogels, films, patches, microneedles, *in situ* gels, ovules, suppositories, implants, or injectable depot systems. This additional formulation step is especially crucial for non-oral delivery, as the ASD must function within a specific dosage form rather than only under simplified dissolution conditions. Consequently, the final therapeutic effect relies on the interaction between two formulation levels: the amorphous dispersion itself and the secondary pharmaceutical base or dosage-form platform in which it is incorporated [21, 22].

The secondary pharmaceutical base or dosage-form platform can either enhance, preserve, or diminish the functional benefits of the ASD. It can increase residence time, spreadability, bioadhesion, tissue contact, hydration, and local retention, thereby extending the time window during which ASD-driven supersaturation can support drug release and tissue exposure [23, 24].

For example, semisolid dermal vehicles may improve skin contact and facilitate drug partitioning into the *stratum corneum* [23]*;* ophthalmic gels can extend precorneal residence time [24]; vaginal and rectal formulations may boost mucosal retention [25, 26]; and injectable bases can be resuspended in an appropriate vehicle and, depending on the route, they may act as depots with controlled release [27]. Therefore, the secondary pharmaceutical base or dosage-form platform should be viewed as an active factor shaping ASD performance, not merely as an inert carrier.

Conversely, the same base can hinder ASD performance if its composition and microstructure do not suit the dispersion. Highly viscous or highly structured vehicles might impede hydration and drug diffusion, delaying release even if the ASD dissolves quickly. Hydrophobic bases could restrict water access to amorphous particles, while aqueous or humid conditions may speed up plasticization, phase separation, or recrystallization during storage [28–30].

Additives like surfactants, cosolvents, salts, polymers, lipids, preservatives, and pH modifiers in the final dosage form can also influence drug–polymer interactions, supersaturation stability, precipitation rates, and drug partitioning into tissues. Thus, the secondary formulation should be viewed as an active factor shaping ASD performance, not just an inert carrier [28, 30].

This interaction is particularly important for translation, quality by design (QbD), and chemistry, manufacturing, and control (CMC) considerations [31, 32]. The key quality attributes of a non-oral ASD-based product include not only the solid-state form, drug–polymer miscibility, residual crystallinity, molecular mobility, and moisture content, but also rheology, microstructure, spreadability, adhesion, syringeability, aerosolization, erosion, swelling, mucoadhesion, and drug release behavior of the final dosage form [33–36].

Assessing the ASD alone may not be enough to predict *in vivo* performance. The final dosage form should be evaluated under biorelevant conditions that mimic the target route, such as limited fluid volume, clearance, tissue barriers, local pH, mucus, tear fluid, skin lipids, or depot environments [37–41].

The secondary pharmaceutical base or dosage-form platform adds an additional translational layer between ASD design and clinical performance. For a non-oral ASD-based formulation to succeed, the amorphous system and the final dosage form must be aligned, ensuring that solid-state stabilization, hydration, release, retention, and tissue exposure occur within the appropriate biological time frame. This highlights the need for ASD development for non-oral routes to go beyond just improving solubility and to embrace a comprehensive product-design strategy [33–35].

For example, pulmonary delivery presents a notable difference. In dry powder inhalation, the ASD can serve as the final drug product itself, eliminating the need to incorporate it into a secondary pharmaceutical base or additional dosage-form platform. This straightforward approach is beneficial because it eliminates an extra formulation step and streamlines the transition from material design to the final dosage form. However, without a surrounding pharmaceutical vehicle to mitigate issues with release, hydration, or stability, the physicochemical and aerodynamic properties of the ASD powder directly influence *in vivo* performance [33].

In parenteral formulations, the injectable vehicle, polymeric matrix, or depot-forming platform plays a crucial role because the ASD might be delivered not as a fully dissolved system but as a sterile suspension, implant, or *in situ*-forming depot [42]. In such scenarios, the surrounding vehicle or polymeric matrix directly influences suspendability, syringeability, injectability, local retention, depot formation, and the drug's post-administration release rate [43]. This is relevant for intramuscular and subcutaneous routes, where ASD can stay at the injection site, acting as a drug reservoir, allowing controlled dissolution, minimizes burst release, and extends systemic exposure [40].

The secondary pharmaceutical base or dosage-form platform should be regarded as an active contributor to performance rather than a passive carrier, as it affects not only release and stability but also local tolerability, sterility, particle control, and the consistency of pharmacokinetic behavior. Therefore, interaction between ASD and formulation must be taken into account, and differences in performance should be expected when distinct pharmaceutical bases or dosage-form platforms are compared during development [40, 42, 43].

### Local Drug Exposure as the Relevant Biopharmaceutical Endpoint

In non-oral administration, the key endpoint is often local drug exposure rather than systemic bioavailability. This distinction is important because simply improving apparent solubility does not ensure a therapeutic benefit [21].

For clinical relevance, the dissolved drug must stay available in a molecularly active form, persist long enough at the site of administration, and establish a sufficient concentration gradient for tissue absorption. Essentially, the therapeutic efficacy of ASDs hinges on whether their physicochemical advantages translate into route-specific exposure rather than merely transient increases in concentration within the donor environment [22, 24].

This idea is particularly critical when the biological interface imposes timing constraints. For instance, in ophthalmic delivery, drainage and tear turnover quickly reduce the time a formulation remains in the eye, making the window for dissolution and absorption very brief. In pulmonary delivery, particles must dissolve and interact with the epithelial lining fluid before clearance mechanisms and regional deposition differences reduce exposure [22, 33].

Conversely, in dermal delivery, the main challenge is not immediate dilution but maintaining a strong thermodynamic driving force for particles to partition into and pass through the *stratum corneum*. The importance of ASD-mediated supersaturation depends on the delivery route itself [21].

### Dissolution in Physiologically Limited Fluid Volumes

A significant challenge in biopharmaceuticals across non-oral routes is that drug release happens in environments with much less fluid than conventional testing setups. For example, the ocular surface has only a small tear volume, the pulmonary epithelium is covered by a thin fluid lining, and both vaginal and rectal routes involve relatively limited and variable fluid conditions. In such cases, dissolution is not just a material characteristic; it is a process influenced by fluid availability, turnover, and local composition [23–26].

This has two key implications for ASD systems. Firstly, a formulation might produce high supersaturation in laboratory tests but behave differently *in vivo* if the available aqueous volume is insufficient to support similar drug release [27].

Secondly, the local fluid composition — such as mucus components, proteins, epithelial secretions, and microbiota-related factors — can influence wetting, diffusion, and drug speciation. For example, in vaginal delivery, mucus, epithelial cell debris, and microbiota-related metabolism can impact formulation behavior and drug distribution [25, 28].

Importantly, the role of the local microbiota remains to be explored, as microorganisms may ferment and biotransform drugs and polymers, potentially leading to instability and uneven biological effects within the system. In the future, this influence could also be taken into account. Consequently, non-oral ASD development requires biorelevant testing environments that more closely mimic the limited and diverse media found *in vivo* [25].

### Tissue Partitioning, Permeation, and Barrier-Controlled Performance

Although ASDs enhance dissolution effectively, drug permeation often remains a separate and limiting step. Biological barriers vary significantly across different routes. For instance, the skin has a dense, lipid-rich *stratum corneum* that restricts penetration; the cornea contains alternating lipophilic and hydrophilic layers that limit transport based on physicochemical properties; the pulmonary epithelium combines thin diffusion distances with clearance mechanisms; and vaginal or rectal tissues are affected by mucus coverage, local tissue physiology, and disease-related factors [21–24].

Biopharmaceutical translation involves more than just transitioning from supersaturation to effectiveness. The success of ASD-mediated solubilization depends on whether the free-drug concentration reaches the target tissue before being diminished by factors such as drainage, mucociliary clearance, dilution, or limited mucosal contact [25, 26].

This aspect is mechanicalistically important in non-oral systems where local exposure results from the dynamic balance between permeation and clearance, rather than dissolution alone. Consequently, tissue partitioning and permeation should be considered jointly as key factors influencing ASD performance, instead of focusing solely on increased solubility [21, 26].

### Route-Dependent Pharmacokinetic and Pharmacodynamic Relationship Considerations for Non-Oral ASDs

The pharmacokinetic and pharmacodynamic relationship (PK/PD) of non-oral ASDs varies widely by route of administration and should not be generalized with a single framework. For systemic non-oral routes such as transdermal, pulmonary, nasal, or certain parenteral routes, ASD effectiveness may depend on plasma exposure metrics such as Maximum Plasma Concentration (Cmax), Time to Reach Maximum Plasma Concentration (Tmax), Area Under the Plasma Concentration–Time Curve (AUC), fluctuation index, and the duration of therapeutic levels. The main question in these cases is whether amorphization and better dissolution lead to more consistent systemic exposure and a meaningful pharmacodynamic effect [44].

For routes that act locally, plasma pharmacokinetics might not be enough or could even be misleading. In dermal, ophthalmic, otological, vaginal, rectal, and subcutaneous applications, the key exposure often concerns the concentration of the active drug in the target tissue rather than systemic bioavailability. Thus, PK/PD evaluations should include local drug retention, tissue partitioning, penetration depth, residence time, and clearance at the site of administration [44].

For instance, in ophthalmic delivery, an ASD-based system should be assessed for its ability to extend precorneal or intraocular exposure and improve outcomes, such as lowering intraocular pressure, reducing inflammation, or combating pathogens [45]. Similarly, for dermal and intradermal routes, PK/PD relevance may relate to drug accumulation in the *stratum corneum*, viable epidermis, dermis, or tumor tissue, as well as local pharmacodynamic indicators such as reduced inflammation, antimicrobial effects, or lesion healing [46, 47].

In parenteral depot systems, such as intramuscular and subcutaneous formulations, the PK/PD relationship is influenced by factors including depot formation, local dissolution, polymer degradation, burst release, and ongoing absorption into the bloodstream. The aim may not be quick supersaturation but rather sustained, consistent drug exposure with minimal peak-trough fluctuations. Consequently, PK/PD evaluation should include long-term plasma concentration profiles, the duration of pharmacological effects, local tolerability, and the correlation between *in vitro* release and *in vivo* performance [48, 49].

Translating ASDs for non-oral routes involves demonstrating that physicochemical benefits—such as amorphization, increased apparent solubility, and supersaturation—translate into meaningful exposure and therapeutic effects relevant to the route [44]. Future research should go beyond simple dissolution or release tests and include biorelevant PK/PD endpoints, such as local tissue exposure, systemic pharmacokinetics when relevant, pharmacodynamic biomarkers, therapeutic outcomes, and safety markers, all tailored to the specific route of administration.

### Route-Dependent Translational Constraints

A major challenge in translation is that no single release profile is ideal for all non-oral routes. For ocular delivery, the formulation must maintain a narrow residence time while remaining clear and tolerable. Pulmonary delivery performance depends on aerosol behavior, regional deposition, dissolution, and epithelial uptake [22, 32].

In dermal applications, longer-lasting local activity and partitioning are often more important than rapid burst release. In vaginal and rectal delivery, factors such as pH variation, mucus dynamics, and microbiota influence local formulation behavior, whereas rectal systems encounter limited fluid availability, positional variability, and inconsistent retention [21, 25, 26].

Similarly, a formulation that performs well in simulated dissolution media for the oral route may not perform effectively if it does not account for other route-specific clearance and retention mechanisms. Designing ASDs with translational relevance requires route-specific performance criteria rather than relying on universal assumptions about improved release [21, 27].

### Predictive Models and Translational Evidence Gaps

A significant challenge in biopharmaceuticals, and especially ASDs, is the lack of translation between drug development, *in vitro* results, and actual *in vivo* effects. Typical release tests often cannot mimic the fluid constraints, barrier structures, clearance processes, and biological complexity seen in non-oral delivery sites [27, 28].

This issue is particularly critical for ASD systems, which rely on temporary supersaturation, localized phase changes, and environment-dependent release mechanisms. Consequently, dissolution data may overstate their therapeutic effectiveness [12, 27].

Recent route-specific studies increasingly regard the importance of combining biorelevant *in vitro* and ex vivo models to better understand formulation behavior. For vaginal delivery, ex vivo or simulated mucus and tissue models are particularly crucial, as microbiota-mucus-epithelial interactions can influence formulation stability and drug transport [28].

Similar issues are relevant for ocular, pulmonary, and dermal delivery, where barriers and clearance mechanisms significantly impact local drug exposure. These findings underscore a key translational point: the critical evidence is not just the ability to generate supersaturation but also whether it can be maintained and effectively converted into tissue exposure under route-specific conditions [21–24].

The key issue is whether the formulation can match its release profile to the limited fluids, tissue barriers, and clearance mechanisms of the target route [28]. In this context, biopharmaceutical performance acts as the link between mechanistic formulation design and therapeutic effectiveness. At this critical juncture, significant gaps in translational evidence persist.

## Route-Specific Evidence and Translational Gaps for Non-Oral Amorphous Solid Dispersions

The current evidence base for ASDs in non-oral delivery routes is uneven. The field is supported by a strong physicochemical and mechanistic rationale and a growing number of formulation and preclinical studies, but it remains characterized by limited route-specific clinical validation.

For this reason, the translational relevance of ASDs should be discussed based on the available evidence for each route rather than assuming a uniform level of knowledge. The translational process status, depending on the route of administration, is summarized in Table 2.

**Table 2:** Route-specific translational status of non-oral ASD systems

Route	Main biopharmaceutical challenge	Representative ASD-based dosage forms	Most advanced evidence available	Representative example	Translational stage	Key evidence gap	References
Transdermal	Sustained permeation across skin	Transdermal patches	Human PK and tolerability studies	Guanfacine ASD transdermal patch	Early clinical	Limited therapeutic outcome validation	[29, 30]
Dermal	*Stratum corneum* barrier, need for local retention	Films, hydrogels, semisolids	*In vivo* pharmacodynamic data (animal models)	Trans-resveratrol ASD in PVP films with anti-inflammatory activity	Preclinical	Lack of human efficacy data	[29, 31]
Pulmonary	Limited fluid volume, deposition–dissolution coupling	Dry powder inhalers	*In vitro* + *in vivo* (animal)	Inhalable cyclosporine A ASD	Preclinical	Lack of ASD-specific clinical inhalation data	[32]–[34]
Ophthalmic	Tear turnover, short residence time	*In situ* gels, microneedles, inserts	*Ex vivo* + *in vivo* (rabbit)	Amphotericin B ASD microneedles	Preclinical	Lack of clinical validation	[22, 35]
Vaginal	Mucus barrier, variable fluid environment	Bioadhesive gels, films	*In vivo* (infected animal models)	Itraconazole ASD thermosensitive gel	Preclinical	Lack of clinical outcomes data	[25, 36]
Rectal	Low fluid volume, variable retention	*In situ* gels, suppositories	*In vivo* PK + histopathology (animal)	Ibuprofen ASD thermosensitive gel	Early preclinical	Scarcity of translational data	[26, 36]
Intravenous	Dilution compatibility, precipitation risk, particle control, tissue distribution	Sterile suspensions, reconstitutable ASD particles, parenteral dispersions	*In vivo* PK and tissue distribution data (animal models)	Curcumin ASD particles administered by tail-vein injection	Preclinical	Lack of clinically validated sterile ASD products; need for dilution, precipitation, and safety control	[48, 49]
Intramuscular	Depot formation, burst-release control, sustained systemic exposure	Polymeric microspheres, sterile depot suspensions, long-acting injectable systems	*In vitro* release + *in vivo* PK/PD data (animal models)	Goserelin/PLGA ASD-based long-acting microspheres	Preclinical	Lack of human studies; need for local tolerability, and reproducible depot effect	[44, 47]
Subcutaneous	Local depot retention, controlled dissolution, prolonged absorption	PLGA implants, injectable depots, polymeric implantable systems	*In vivo* release data after subcutaneous administration (animal models)	Fenretinide/PVP ASD incorporated into PLGA millicylindrical implants	Preclinical	Limited route-specific PK/PD, injectability or implantation data, and local tissue tolerability studies	[44, 47]
Intradermal	Bypassing *stratum corneum*, dermal deposition, local tissue exposure	Coated microneedles, dissolving microneedles, ASD-loaded microneedle matrices	*In vitro* + *in vivo* pharmacodynamic data (animal models)	Effervescent cannabidiol ASD-doped dissolving microneedles for melanoma therapy	Preclinical	Lack of clinical validation; need for reproducibility, local safety, and human efficacy data	[44, 47]

PD: Pharmacodynamics; PK: Pharmacokinetics; ASD: Amorphous solid dispersion

### Dermal and Transdermal Delivery: The Strongest Non-Oral Evidence Base, Still Mostly Preclinical

Among non-oral routes, dermal and transdermal delivery are among the most advanced for amorphization-based methods. This is plausible mechanistically because increasing apparent solubility and maintaining thermodynamic activity can enhance drug partitioning into the *stratum corneum* and support local tissue exposure [27]. Recent studies on skin delivery confirm that skin barrier properties remain the main limitation, and formulation design must be tailored to whether the aim is local dermal retention or deeper transdermal transport [29].

Recent evidence indicates that the translational maturity of ASD systems varies between transdermal and cutaneous uses. In the transdermal area, ASD formulations have advanced toward clinical testing. For example, in a study by Hou, Zhao & Ding (2024) [30], a guanfacine transdermal patch prepared using a ASD method was evaluated in human pharmacokinetic trials, assessing adhesion, skin irritation, and sensitization. This suggests that the approach is nearing clinical application and has reached a higher stage of development.

In contrast, ASD applications for local cutaneous delivery remain at the preclinical stage, despite important progress. A representative example is a study performed by our group [31], in which trans-resveratrol ASDs were incorporated into povidone (PVP)-based films, which were thoroughly characterized physicochemically and evaluated in an *in vivo* mouse ear cutaneous inflammation model, demonstrating pharmacodynamic activity. Such studies indicate that cutaneous ASD systems have evolved beyond formulation feasibility and now include biologically relevant *in vivo* validation.

These findings reveal a disparity in the progress of translational development across different routes. While transdermal ASD systems are showing clinical promise, cutaneous formulations are still in an intermediate phase, with successful incorporation into functional dosage forms and preclinical validation, but lacking substantial human data [29].

The key challenge in dermal translation is not the absence of a rationale but the limited high-quality human evidence demonstrating that increased release or permeation leads to improved therapeutic outcomes, such as faster symptom relief, less severe lesions, lower doses, or enhanced tolerability.

### Pulmonary Delivery: Strong Mechanistic Rationale, Limited ASD-Specific Translation

Pulmonary delivery, especially via dry powder inhalers, offers a clear mechanistic rationale for using ASDs. Since drug dissolution occurs in a very limited fluid environment with rapid clearance, ASD-driven supersaturation can provide a direct biopharmaceutical benefit by speeding up local drug availability after deposition. However, this route also presents a significant translational challenge, as pulmonary success relies on managing amorphous-state stability, aerosolization, particle engineering, and dissolution in the epithelial lining fluid simultaneously [32, 33].

To date, the most advanced evidence for pulmonary ASDs remains preclinical. Early but representative studies include the study by Onoue and colleagues (2009) [34], which evaluated inhalable cyclosporine A ASDs *in vitro* and *in vivo* in rats. In short, pulmonary ASD systems can move beyond simple solid-state characterization and reach biologically and pharmaceutically meaningful testing. Nevertheless, robust ASD-specific clinical studies in inhalation remain scarce.

This directness is advantageous from a structural standpoint, as powders can be administered via the pulmonary route, which does not necessarily require a secondary pharmaceutical base, but it also means that suboptimal release, instability, or moisture sensitivity are less likely to be compensated by formulation-level buffering [31, 32]. Therefore, pulmonary ASDs may be considered structurally less dependent on secondary pharmaceutical bases, yet demanding in terms of translational reliability.

### Ophthalmic Delivery: High Need, but Evidence Remains Primarily Formulative and Preclinical

Ophthalmic delivery presents a compelling reason to use ASDs, primarily because traditional eye drops face significant limitations due to rapid tear turnover, drainage via the nasolacrimal duct, and short precorneal contact times, which result in low bioavailability. In this context, ASDs are particularly valuable when incorporated into secondary ophthalmic formulation bases, such as *in situ* gels, to enhance contact time with the ocular surface [22].

However, the clinical translation of ophthalmic ASDs remains at the preclinical stage. For instance, one of the most advanced preclinical cases is in the study made by Mahfud *et al*. (2023) [35], with the use of amphotericin B ASDs in dissolving ocular microneedles for fungal keratitis, advancing to ex vivo corneal permeation, ocular irritation tests, and *in vivo* antifungal evaluations in rabbits, outperforming traditional eye drops and ointments.

Currently, ophthalmic ASDs have evolved beyond simple physicochemical proof of concept and can be integrated into route-appropriate dosage forms with meaningful preclinical performance. Nevertheless, the field still lacks robust ASD-specific clinical evidence, suggesting that ophthalmic applications currently lie between formulation development and clinical consolidation. The main concern is not the theoretical foundation but the absence of comprehensive *in vivo* and clinical comparative research.

### Vaginal Delivery: Promising Local Application, but Evidence is Still Thin

Vaginal delivery is a promising yet still developing area for ASD systems. Currently, the strongest evidence is preclinical. One of the most advanced examples involves the study by Permana and collaborators (2021) [36], which reported bioadhesive, thermosensitive *in situ* vaginal gels containing itraconazole ASD gel flakes that exhibited improved solubility, longer vaginal tissue retention, and strong antifungal activity in infected animal models.

In this sense, vaginal ASD systems have moved beyond basic formulation testing and now have preclinical evidence. Nevertheless, the field still needs robust clinical trials to confirm improvements in symptom relief, recurrence prevention, tolerability, and patient adherence over conventional vaginal creams and gels [37].

### Rectal Delivery: Conceptually Relevant, but the Weakest Route-Specific Evidence Base

Rectal delivery is still among the least developed routes. While it is an attractive option for poorly soluble drugs due to limited fluid volume, low enzymatic activity, and the potential to bypass first-pass metabolism [26], evidence for rectal ASDs remains limited and preclinical.

One notable example is a study performed by Liu and colleagues (2018) [37]. The authors developed a thermosensitive *in situ* gel based on ibuprofen ASD, advancing from formulation studies to include *in vivo* pharmacokinetics in rabbits and rectal histopathology, demonstrating higher plasma levels than traditional suppositories without irritation. Studies indicate that rectal ASDs are feasible and can be formulated into suitable dosage forms, but the field lacks strong evidence of their clinical benefits in humans.

### Parenteral Delivery: A Special Case for ASD Administration That Remains in Preclinical Phase

Parenteral administration comprises the intravenous, intramuscular, subcutaneous and intradermal routes. This is a unique non-oral administration because its biopharmaceutical needs differ significantly from those of epithelial or mucosal delivery, which does not necessarily require administration as fully dissolved systems [48, 49].

Depending on the route and therapeutic objective, particles may be resuspended, dispersed in an aqueous or non-aqueous vehicle, and administered as sterile suspensions, particularly for intramuscular or subcutaneous depot applications [50]. In this context, the ASD can act not only as a solubility-modifying platform, but also as a solid-state reservoir from which the drug is gradually released after administration [51].

This is especially relevant for intramuscular delivery, where the formulation may remain within the muscle tissue and form a local depot, allowing controlled dissolution, prolonged release, and sustained systemic exposure [51, 52]. For parenteral ASD-based systems, the relevant performance attributes extend beyond amorphization and apparent solubility to include particle size distribution, suspendability, syringeability, injectability, sedimentation behavior, local tolerability, depot formation, and reproducible long-term release [52, 53].

In this context, it is important to interpret the role of amorphization carefully. For traditional polymer-based ASDs, the primary goal is to achieve and sustain supersaturation upon contact with an aqueous environment [54]. However, during parenteral administration, uncontrolled supersaturation can be problematic if it leads to rapid precipitation upon dilution in infusion fluids or blood [55]. Therefore, key quality attributes include solid-state stability prior to reconstitution, rapid and consistent reconstitution, compatibility with infusion media, prevention of crystallization or aggregation during administration, acceptable osmolality and pH levels, safe excipients, sterility, and effective control of particle size [40].

Parenteral ASDs are still in the preclinical phase rather than fully established ASD clinical platforms. Moving toward clinical use will demand more than just proving amorphization or enhanced apparent solubility. Important considerations include injectability, syringeability, particle size distribution, depot formation, local tolerability, burst-release control, polymer degradation, *in vitro*–*in vivo* correlation, sterilization compatibility, and consistent pharmacokinetic performance over time.

#### Intravenous Administration

The intravenous route remains an underexplored area for classical ASDs. Existing research indicates that polymer-based ASDs intended for intravenous use are mainly at the formulation development or preclinical proof-of-concept stages [56]. Unlike epithelial routes, this route represents a special case in which uncontrolled supersaturation may become undesirable. Consequently, the main formulation challenge is not enhancing permeation but ensuring the product is sterile, easy to administer, physically stable, and compatible with dilution, all while maintaining drug availability without precipitation or excessive particulate formation during reconstitution, dilution, and infusion [40, 54].

Kato *et al*. (2023) [57] examined curcumin dispersions of various particle sizes in rats, administering them via the tail vein. They measured plasma levels of curcumin and its glucuronide post-injection. Notably, larger particles raised plasma levels of unaltered curcumin and seemed to decrease its conversion to curcumin glucuronide. The study also raised concerns about tissue deposition, especially in the lung and liver, for bigger particles. This example shows that intravenous ASDs are still in early preclinical research, and successful translation depends on more than improved solubility; it requires careful control of particle size, dilution, metabolism, tissue distribution, and safety.

#### Intramuscular Administration

Intramuscular administration is a unique parenteral route for ASDs because the formulation typically remains at the injection site, acting as a depot rather than dissolving immediately into the tissue. In this route, the main goals are not only to improve drug solubility but also to manage local dissolution, depot structure, tissue retention, burst release, and long-term systemic levels [58]. Among the available studies, ASDs are mainly examined as technological tools to enhance drug distribution within depot systems and to control release from polymeric matrices [59].

Ren *et al*. (2022) [58] reported that a goserelin and PLGA ASD was first prepared by hot-melt extrusion and subsequently dissolved or dispersed in dichloromethane to produce long-acting PLGA microspheres by an oil-in-water method. This strategy was proposed to improve the distribution of the peptide within the polymeric matrix and to reduce the initial burst release commonly observed in peptide-loaded PLGA microspheres.

The optimized formulation revealed that goserelin was molecularly dispersed in an amorphous form within the microspheres, with no evidence of aggregation. This ASD approach significantly reduced the initial burst release compared to microspheres produced by the traditional solid-in-oil-in-water method. Additionally, the formulation enabled sustained drug release, achieving nearly complete release by 45 days. *In vivo* studies demonstrated that the microspheres maintained relatively stable serum goserelin levels for approximately 35 days and elicited a pharmacodynamic response, as evidenced by suppression of testosterone secretion in male rats [40]. This example supports the potential of ASD-based approaches for long-acting injectable systems, while also showing that the field remains at a preclinical stage.

#### Subcutaneous and intradermal routes

Subcutaneous and intradermal routes are two additional parenteral routes in which ASDs can be beneficial. In subcutaneous administration, the formulation typically stays near the injection site, forming a local depot that manages drug dissolution, release, and systemic absorption over time [60]. Consequently, ASDs are meant to enhance the inclusion and release of poorly soluble drugs from biodegradable matrices, implants, or injectable depot systems [61].

Nieto, Mallery, and Schwendeman (2020) [61] reported a relevant example involving fenretinide and povidone (PVP) ASD integrated into PLGA millicylindrical implants. This approach aimed to address fenretinide's high hydrophobicity and enhance its release from PLGA-based long-acting depots. The optimized formulation significantly boosted water solubility, and adding a plasticizer further improved drug release. Crucially, *in vivo* evaluation in rats via subcutaneous injection showed sustained, nearly complete drug release over about a month.

Intradermal delivery is closely associated with microneedle-based methods. Here, ASDs can either be coated onto microneedles or integrated into dissolving microneedle matrices [62]. This enhances the apparent solubility of poorly water-soluble drugs and aids their deposition into the viable epidermis or dermis [63]. This technique is especially useful for local treatments, vaccination strategies, and skin cancer models, as microneedles can bypass the *stratum corneum* and deliver formulations directly into skin microenvironments [64].

Shi *et al*. (2023) [64] developed dissolving microneedles doped with effervescent cannabidiol ASD and dispersed in PVP for melanoma treatment. In this system, cannabidiol ASD was incorporated into the microneedles alongside effervescent components such as calcium carbonate and sodium bicarbonate. Once inserted into the skin, the system released CO₂ bubbles, which actively boosted both skin permeation and tumor penetration of cannabidiol. In addition to enhancing local delivery, the formulation modulated the tumor microenvironment and boosted antitumor activity through mechanisms including TRPV1 activation, calcium influx, inhibition of the NFATc1–ATF3 pathway, and induction of apoptosis. The system was tested both *in vitro* using a murine melanoma cell line B16-F10 and *in vivo* in a male mouse melanoma model, demonstrating improved anti-melanoma effects.

### Overall Translational Reading of the Evidence

The existing non-oral ASD literature presents an uneven outlook. ASD-specific transdermal systems currently represent the most advanced route with reported clinical pharmacokinetic evaluation, though the cutaneous route has solid evidence in the *in vivo* animal models. The same can be said for pulmonary delivery, which has already shown evidence in animal models. Ophthalmic delivery addresses a significant need but remains primarily in preclinical research. Vaginal delivery shows promise, backed by solid mechanistic and engineering rationale, but its practical application faces challenges and has limited validation, whereas rectal delivery is the least developed in practice [65–70].

This distribution of evidence carries significant implications for the field. The primary challenge no longer lies in explaining why ASDs might be effective in non-oral systems. Instead, the key obstacle is the lack of route-specific translational evidence linking the benefits of the amorphous state to meaningful clinical outcomes.

Future research should focus on comparative studies, biorelevant performance models, and route-oriented *in vivo* validation, rather than just rehashing generic claims of enhanced solubility. The field remains dominated by physicochemical and formulation-level evidence, with limited route-specific translational validation.

## Retrospective Overview and Recent Advances in Non-Oral ASD Formulations

The development of non-oral ASDs has evolved from simple solubility-enhancing systems toward more route-specific and functionally integrated dosage forms. Early studies mainly focused on demonstrating amorphization, improved apparent solubility, faster dissolution, or enhanced release of poorly water-soluble drugs. In this initial stage, the ASD was often evaluated as an isolated material, and its performance was primarily assessed using physicochemical parameters such as crystallinity reduction, drug–polymer miscibility, dissolution rate, and supersaturation behavior; when evaluated more complexly, these assessments were almost restricted to the oral route [71, 72].

More recent studies have shifted toward incorporating ASDs into final non-oral dosage forms designed to address specific anatomical and physiological barriers. In dermal and transdermal delivery, ASDs have been incorporated into films, patches, semisolid bases, and microneedle systems to improve drug partitioning, local retention, or systemic permeation through the skin [30].

In ophthalmic delivery, ASDs have been combined with microneedles or *in situ* systems to overcome short precorneal residence time and improve corneal or intraocular exposure [73]. In vaginal and rectal delivery, thermosensitive, bioadhesive, or mucoadhesive bases have been used to prolong local residence and sustain drug release under limited fluid conditions [65, 66].

In pulmonary delivery, amorphous powders have been explored as inhalable systems in which the ASD may function directly as the final dosage form, making particle engineering, aerodynamic behavior, and moisture stability central determinants of performance [74, 75]. For parenteral delivery, the aims are sterility, absence of pyrogenic substances, and the possibility to formulate powders for extemporaneous suspensions for injection [40].

A further step in the evolution of the field is the use of ASDs as technological intermediates in more complex systems, including dissolving microneedles, biodegradable implants, injectable depots, and hybrid solubilization platforms. In these cases, the ASD is not necessarily the final product itself, but contributes to drug distribution within a polymeric matrix, modulation of release, reduction of burst effect, or maintenance of drug availability after administration. This is particularly relevant for parenteral depot systems, in which ASD-based intermediates may support controlled dissolution and prolonged systemic exposure rather than immediate supersaturation [61, 64].

In this sense, this retrospective reading indicates that the field has progressed from proof-of-concept amorphization studies to more sophisticated, route-oriented formulation strategies. However, most non-oral ASD systems remain at the formulation-development or preclinical stage. The main translational gap is no longer only about whether they can improve solubility or release, but also about whether these physicochemical benefits can be reproducibly translated into route-specific pharmacokinetic, pharmacodynamic, safety, manufacturability, and patient-relevant advantages.

## Pharmaceutical and Translational Barriers for Non-Oral ASDs

Despite solid mechanistic support and growing formulation and preclinical evidence, the clinical use of ASDs beyond oral delivery remains mostly in formulation-driven or preclinical phases. This is not mainly due to a lack of physicochemical understanding, but because translating formulation benefits into dependable, route-specific therapeutic outcomes is difficult.

Consequently, barriers to applying these findings include issues of predictability, complex formulation requirements, physiological differences, and regulatory challenges. In this context, studies and commercial products may not always disclose the presence of ASDs in their formulations; rather, they report only the presence of polymers and surfactants, without clarifying whether these excipients form an amorphous dispersion or merely serve as conventional formulation components.

### Limited Predictive Power of Conventional In Vitro Models

A major challenge in adapting ASD-based systems for non-oral applications is that traditional *in vitro* testing methods often lack predictive accuracy. Many dissolution and release tests were initially developed for oral delivery and are typically performed under sink conditions with large volumes of fluid [65, 66]. However, these conditions do not accurately reflect the physiological environments of non-oral routes, which typically involve limited fluids, dynamic fluid movement, and diverse biological interfaces [40, 41].

Improvements in the *in vitro* dissolution and release do not always translate into better *in vivo* results. This limitation is particularly relevant for ASD systems, which rely on transient supersaturation, changes in phase behavior, and environmental factors. Without biorelevant models that account for these dynamics, there is a risk of overestimating the predictive value by only *in vitro* release or dissolution data [67].

### Disconnect Between Supersaturation and Therapeutic Relevance

While supersaturation is often considered a main benefit of ASDs, its clinical significance remains insufficiently established outside oral applications. Achieving high apparent solubility does not guarantee improved therapeutic outcomes if the dissolved drug is rapidly cleared, fails to penetrate the target tissue, or is converted into non-bioavailable forms [67].

This disconnect is especially clear in routes where local exposure is influenced by competing processes, such as clearance and barrier resistance. In these situations, the duration of supersaturation and its capacity to sustain a therapeutically effective free-drug level are more critical than the magnitude of supersaturation itself [74]. Future research should go beyond simply reporting increases in apparent solubility and instead aim to link supersaturation behavior to measurable pharmacodynamic or clinical outcomes.

### Regulatory and Translational Evidence Requirements

From a regulatory standpoint, translating ASDs into non-oral drug forms involves more than just showing improved apparent solubility or faster *in vitro* release. Regulatory approval ultimately depends on demonstrating that the formulation can reliably deliver a performance profile that is both reproducible and clinically relevant, within a well-managed pharmaceutical quality system [75–78].

In this context, non-oral ASD systems face two regulatory challenges: they must address the complexity of amorphous, metastable materials and meet route-specific requirements for locally acting, inhaled, semisolid, mucosal, or sterile formulations [79–82]. Additionally, many studies do not adequately consider the relationship between ASD and the secondary formulation base, thereby limiting their practical applicability and keeping many at the physicochemical proof-of-concept level.

For ASD-based products, these principles suggest that the control strategy should extend beyond just composition to include critical material attributes and process parameters. These factors influence solid-state form, residual moisture, phase stability, dissolution behavior, and, where applicable, particle properties [79–82]. This is especially vital because the clinically relevant performance of an ASD may rely on features that traditional release testing does not fully capture.

A significant regulatory challenge arises because non-oral ASD systems often fall into categories considered complex products. For topical and other local applications, regulators increasingly depend on physicochemical and structural analyses, along with *in vitro* release and permeation tests, to demonstrate performance and equivalence [79, 81].

However, ASD-based systems pose greater challenges, as their performance can depend on factors beyond the final dosage form, such as metastable phenomena, including maintaining supersaturation, hydration-driven phase shifts, and crystallization at or near the administration site [81, 82].

Consequently, routine quality assessments may be inadequate unless complemented by biorelevant methods that accurately reflect route-specific formulation behavior. More biorelevant *in vitro* characterization strategies may be critical to reduce this translational gap [79, 82].

Regulatory standards for pulmonary products can be particularly stringent, especially when the ASD serves as the final dry powder inhaler dosage form. As a result, regulators are likely to require thorough control of factors such as amorphous-state stability, particle engineering, aerosol performance, moisture sensitivity, and manufacturing consistency [81].

For ophthalmic, parenteral, and other sterile-route products, an additional regulatory requirement applies. In these cases, developers must control ASD-related material attributes and also ensure sterility. The challenge is not only to demonstrate that an ASD enhances drug release, but also to show that this benefit is maintained within a dosage form and packaging suitable for the target route [82].

Parenteral formulations represent a distinct category within the non-oral ASD landscape, as they must meet strict requirements for sterility, pyrogen/endotoxin control, and particulate safety [83]. Intramuscular, subcutaneous, and intradermal administration, unlike delivery across epithelial or mucosal absorption barriers, do not necessarily require the drug to cross an absorption barrier at the site of administration, nor do they always require the drug to be administered as a fully solubilized formulation; rather, suspensions can be used to form depots within the tissue [84]. Intravenous administration, however, should be designed to ensure rapid and reproducible drug availability while avoiding precipitation, aggregation, or formation of unsafe particulate matter after reconstitution, dilution, or contact with blood [85].

In summary, the regulatory gap for non-oral ASDs goes beyond simply lacking route-specific guidelines. The key challenge is to align current regulatory frameworks for complex formulations with the distinctive performance characteristics of amorphous systems. Future progress should likely involve developing control strategies tailored to each route of administration, enhancing biorelevant performance testing, and establishing stronger links between physicochemical data and real-world clinical outcomes.

## Patent Landscape and Industrial Translation of Non-Oral ASD-Relevant Products

While ASD technology is well established for oral medications, its application to non-oral forms is less clearly documented. A key challenge in identifying marketed non-oral ASD-based products is that regulatory labels, product details, and prescribing information often do not specify whether the active ingredient is an ASD. Frequently, these products only mention the active compound, excipients, polymeric carriers, or manufacturing methods, without clarifying whether the drug is molecularly dispersed, amorphous, or stabilized in a polymer matrix. Consequently, identifying products containing ASDs, orally or not, based solely on their drug and excipient profiles can lead to overinterpretation.

Therefore, this review took a cautious approach, focusing on patent documents that explicitly mention ASD systems designed for non-oral use (Table 3) [86–91]. Patent documents are valuable complementary sources of industrial and technical information because they often reveal formulation rationale, the physical state of the drug, polymeric carriers, manufacturing methods, and intended routes of administration more directly than product labels do. Nonetheless, patent data should be interpreted carefully, as it reflects technological development and intellectual property activity but does not guarantee clinical approval, commercial availability, therapeutic advantage, or successful market entry.

**Table 3:** Patent evidence of non-oral ASD technologies and their intended therapeutic applications

Route/application	Patent or patent family	Therapeutic indication	Drug/bioactive	Polymer/carrier	Claimed SD/ASD evidence	References
Transdermal	US10130589B2/related UCB patents	Parkinson’s disease and restless legs syndrome	Rotigotine	Polyvinylpyrrolidone; silicone pressure-sensitive adhesive matrix	Claims a SD comprising PVP and non-crystalline rotigotine free base; also claims a transdermal therapeutic system comprising this SD	[86]
Transdermal/microneedle	WO2016155891A1	Psoriasis and inflammatory skin disorders	Betamethasone dipropionate and calcipotriol	Water-soluble polymer matrix	Describes the active ingredients as glass solutions molecularly dispersed in the polymer matrix	[87]
Transdermal/microneedle	CN109528695B	Pain and inflammation	Celecoxib	Microneedle polymer matrix	Describes formation of celecoxib SD microneedles after solidification of the microneedle liquid	[88]
Intradermal/microneedle	CN111035628A	Hypertrophic scars/scar treatment	Tanshinone IIA	Self-dissolving microneedle matrix	Describes a drug-loaded microneedle layer formed by mixing tanshinone IIA SD with the matrix material	[89]
Ophthalmic	WO2019053466A1	Ocular diseases requiring sustained local drug delivery	Dexamethasone or other ophthalmic active agents	Polymeric microspheres; PLGA-based systems in some embodiments	Describes ophthalmic compositions in which the active agent may be present in crystalline or ASD form to tailor release	[90]
General solid dispersion manufacturing	EP0617612B1	Poorly soluble drugs requiring improved delivery	Various active ingredients	Inert carrier or matrix; extrusion-compatible carrier systems	Defines SD as active ingredient dispersed in an inert carrier or matrix in the solid state and describes manufacturing approaches	[91]

Patent documents were included only when they explicitly described the presence of SD or ASD. These examples should be interpreted as evidence of technological and industrial interest rather than proof of clinical approval or marketed ASD-based products

## Positioning Solid Dispersions Among Solubility-Enhancing Technologies

Within the broader field of methods to enhance biopharmaceutical properties, ASDs represent one relevant approach by improving poor drug dissolution by altering the solid state rather than relying on colloidal stabilization or complex self-assembly. This difference is significant from a practical perspective, as the usefulness of ASDs depends not only on their capacity to enhance apparent solubility but also on the extent of formulation complexity needed to sustain that benefit in real-world applications [92]. In Table 4, the ASDs are compared to the other available technologies.

**Table 4:** Positioning ASDs among biopharmaceutical property-enhancing technologies

Technology	Primary mechanism	Main strengths	Main limitations	Translational complexity	Most suitable scenario	References
Amorphous solid dispersions	Solid-state modification and supersaturation	Strong dissolution-driving force, industrially established methods	Metastability, crystallization risk	Moderate	When poor performance is driven by solid-state limitations	[48, 49]
Nanoparticles	Colloidal stabilization and nanoscale size	Targeting, mucus interaction, controlled biodistribution	Aggregation, physicochemical instability	High	When advanced delivery functions are needed	[48, 50, 51]
Nanocrystals	Size reduction while maintaining crystallinity	High drug loading, increased dissolution rate	Limited increase in thermodynamic activity	Moderate	When the dissolution rate is limiting	[48, 52]
Cocrystals	Crystal engineering with coformers	Improved stability and manufacturability	Moderate solubility enhancement	Moderate	When a crystalline strategy is preferred	[48, 53]
Cyclodextrins	Inclusion complexation	Improved solubility and stability, mature regulatory profile	Limited drug loading, equilibrium-dependent	Low–moderate	When formulation tolerability is critical	[48, 54]
Polymeric micelles	Self-assembled nanostructures	Solubilization + targeting + co-delivery	Dilution sensitivity, structural instability	High	When advanced drug delivery is required	[55, 56]
Lipid-based systems	Drug solubilization in lipid matrices	Effective for lipophilic drugs, absorption enhancement	High formulation dependence	Moderate-high	When lipophilicity is the main limitation	[48, 57]
Emulsions	Drug solubilization in dispersed oil or interfacial phases	Effective for lipophilic drugs, improved spreadability and tissue contact	Physical instability, droplet-size control, phase separation, oxidation, and preservation challenges	Moderate–high	When a dispersed liquid or semisolid system is acceptable	[103]-[105]
Solvents and cosolvents	Direct drug solubilization by solvent environment modification	Simple formulation, rapid drug availability, established clinical use	Irritation, toxicity, precipitation after dilution, limited tolerability and packaging	Low–moderate	When immediate solubilization is required and excipients are route-compatible	[110, 111]

### Nanoparticles and Encapsulation Systems

ASDs offer specific benefits over nanoparticle-based and other encapsulation systems for solubility enhancement, particularly in formulation and translation. Both systems aim to boost drug bioavailability, but differ fundamentally in their structures [93].

Most types of nanoparticles are colloidal systems stabilized at interfaces, making them prone to aggregation, Ostwald ripening and instability, depending on formulation or biological conditions. Ostwald ripening refers to the growth of larger particles or droplets at the expense of smaller ones, driven by the higher solubility and dissolution tendency of smaller particles followed by molecular diffusion and redeposition onto larger particles. Conversely, ASDs are solid-state systems in which the drug is molecularly dispersed within a polymer matrix, potentially reducing some colloidal stability concerns, but introducing other challenges, such as humidity, recrystallization, and the sustained amorphous stability [94].

This distinction has significant implications for pharmaceutical development. ASDs leverage well-established manufacturing methods such as spray drying and hot-melt extrusion, which are widely used in industry. These features can ease scale-up, enhance reproducibility, and aid regulatory approval [93].

In contrast, nanoparticle systems typically require more sophisticated formulation techniques, stricter control of physicochemical properties, and more detailed characterization to ensure stability and performance [94]. Compared with others, ASDs may offer manufacturing advantages in some cases, though this depends on product complexity and route-specific requirements.

A key difference is in how they are integrated into dosage forms. ASDs naturally lend themselves to solid forms such as powders, suppositories, and films, which can be compatible with solid or semisolid dosage forms but usually require route-specific optimization [68, 69]. Conversely, nanoparticle systems often require a secondary carrier or dispersion medium, which can destabilize the system, leading to agglomerations or aggregations [94, 95].

However, these benefits should be viewed in context. Encapsulation systems offer advantages over ASDs, including enhanced tissue targeting, improved interactions with biological barriers such as mucus, and more controlled biodistribution. They may also reduce the drug costs and make it easier to modulate the drug release [94–96].

Choosing between these systems is not about which is better. ASDs may be suitable for applications in which solid-state dissolution is the main limitation and established manufacturing technologies can be applied, though nanoparticle systems might be preferable for more advanced delivery functions [95, 97]. From a translational standpoint, although complex systems can offer additional features, they often entail greater development challenges and greater uncertainty during clinical translation.

### Nanocrystals and Cocrystals

A key comparator is drug nanocrystal technology. Nanocrystals and nanosuspensions are adaptable systems that can be incorporated into various dosage forms, such as hydrogels and microneedles. Their primary benefit is increasing surface area and dissolution rate while keeping the drug's crystalline form, often with high drug loading [98].

ASDs, on the other hand, which depend on partial or complete loss of crystallinity and on polymer-assisted stabilization of a higher-energy state, are advantageous when maintaining amorphization is important for enhancing bioavailability. They are better suited when solubility and dissolution are mainly limited by solid-state factors and when a molecularly dispersed drug state is needed to enhance release performance [95, 98].

Cocrystals offer a distinct approach by enhancing performance while remaining crystalline. This can provide benefits in solid-state order, and in some cases, improve manufacturability and stability. However, cocrystals usually rely on suitable coformers and may provide smaller or more controlled solubility changes in some cases [99].

In this regard, cocrystals may be preferable when a more thermodynamically stable crystalline form is desired, whereas ASDs may be better suited if the goal is to achieve a stronger solubilization drive via a metastable amorphous state [94, 99].

### Cyclodextrins

Cyclodextrins enhance aqueous solubility, improve chemical stability, mask undesirable properties, and, in some advanced derivatives, even promote mucoadhesion, permeation, or sustained release. Their benefits stem from molecular inclusion rather than amorphization [100].

They are an important comparator for ASDs because they enhance drug performance through a different mechanism. Instead of increasing thermodynamic activity via amorphization and metastable supersaturation, cyclodextrins may improve apparent solubility by forming reversible inclusion complexes. This can also enhance chemical stability, mask unpleasant tastes, and support various formulations used in clinical practice [100].

As a result, cyclodextrin-based systems have a translational advantage, backed by a mature regulatory and development landscape. However, some points to consider are that their effectiveness depends on equilibrium-dependent complexation and cavity-dependent drug loading, and that higher total solubility does not always translate into an increase in free drug available for permeation, while ASDs may be more challenging due to the sometimes-needed use of solvents and surfactants instead of just water, as most of the cyclodextrin inclusions [92, 100].

Cyclodextrins and ASDs are not interchangeable strategies: cyclodextrins are particularly useful when local solubilization, excipient functionality, or formulation tolerability are key, whereas solid dispersions may be better for overcoming solid-state limitations and increasing the dissolution driving force.

### Polymeric Micelles

Polymeric micelles are another key comparison point for ASDs because they enhance drug performance through self-assembled nanoscale structures rather than altering the solid state. Their main benefit is the ability to combine solubilization with additional delivery features, such as targeting, stimulus responsiveness, and co-delivery of multiple drugs [101].

However, this self-assembled nature also makes polymeric micelles highly sensitive to dispersion conditions and the need to maintain micellar integrity during physiological dilution and biological exposure [102]. In contrast, ASDs are solid-state systems that do not depend on preserving a colloidal nanostructure to deliver their benefits [92].

Thus, polymeric micelles may be especially useful when advanced delivery functions are needed, while ASDs may be more suitable when the main limitation is solid-state dissolution rather than nanoscale delivery, for overcoming issues related to limited dissolution and drug release in the solid state. While ASDs typically do not match the delivery capabilities of micelles, they can be a simpler solution when the main challenge is solid-state-limited dissolution rather than targeted delivery [93, 102].

### Emulsions and Lipidic Systems

ASDs exhibit distinct translational profiles compared to emulsions and lipid-based systems. Lipid formulations are particularly advantageous for highly lipophilic molecules, as they maintain the drug in solution within a lipid environment and can enhance its absorption by solubilizing it [103].

Emulsions and lipid-based systems are a well-established alternative, particularly for lipophilic drugs. They work by incorporating the drug into an oil phase or interfacial environment, enhancing solubilization, spreadability, tissue contact, and sometimes enabling controlled release. These systems are especially effective for topical, parenteral, ocular, and mucosal delivery when a dispersed liquid form is suitable. However, emulsions also face challenges such as physical instability, droplet-size management, phase separation, oxidation, microbial preservation issues, sterilization difficulties, and variability in drug partitioning between phases and sometimes requiring additional dosage forms [103–105].

Conversely, ASDs are generally chosen to turn compounds amorphous, where poor performance primarily results from crystal lattice energy and slow dissolution rather than nanoscale delivery. Thus, ASDs align more closely with solid pharmaceutical design principles, whereas lipid systems are better suited to encapsulate lipophilic compounds in lipophilic secondary bases or in nanostructured systems [68, 77].

### Solvents and Cosolvents

Systems such as solvents, co-solvents, surfactants, emulsions, and encapsulation techniques are still commonly employed rather than ASD because they maintain the drug in solution or dispersion without requiring amorphization. These methods are especially useful when immediate drug availability, high local concentrations, or routes accepting liquid or semisolid carriers are important [106].

Solvents and co-solvents are among the simplest methods to enhance apparent drug solubility. They offer benefits such as ease of formulation, rapid drug availability, straightforward manufacturing, and extensive clinical use. However, their application can be limited by issues such as local irritation, precipitation upon dilution or administration, limited drug-loading capacity, and incompatibility with certain tissues, packaging materials, or storage conditions. Consequently, while solvent-based systems can be very effective, their successful implementation depends heavily on route-specific safety considerations and on how they behave upon dilution [106–108].

Compared to these methods, ASD uses a different approach: it modifies the drug's solid state and stabilizes a high-energy amorphous form within a carrier matrix. This can enhance apparent solubility, dissolution rate, and thermodynamic activity without necessarily needing large amounts of organic solvents or lipid phases in the final product [69].

### Synergistic Strategies Combining ASDs with Other Solubility-Enhancing Technologies

While ASDs are often compared with other solubility-enhancing methods, these strategies should not be seen as mutually exclusive. In many non-oral cases, the best formulation approach might involve combining amorphization with other technologies, especially when poor solubility is just one of multiple challenges impacting therapeutic effectiveness. In such scenarios, ASDs can deliver a high-energy amorphous drug form and enhance apparent solubility or supersaturation, while complementary methods may aid retention, permeability, stabilization, controlled release, mucoadhesion, tissue targeting, acceptability, or therapeutic adherence [69].

For example, ASDs can be combined with cyclodextrins to improve wettability, reduce crystallization, or increase drug solubility upon hydration [109]. Surfactants and polymeric micelles may help sustain supersaturation and prevent precipitation by stabilizing drug-rich colloidal species during dissolution [110, 111].

Lipid-based systems might provide an additional solubilizing environment for highly lipophilic drugs, while ASD-based particles or intermediates can enhance drug loading [103–105]. Likewise, nanocrystals and cocrystals could be preferred for high drug loading and crystalline stability, whereas hybrid systems with amorphous regions and particulate carriers can be useful for both dissolution enhancement and controlled release [112, 113].

This synergistic approach is especially important for non-oral delivery methods, where formulation performance hinges on more than just solubility. The final product must also contend with usually limited fluid volumes, clearance, tissue barriers, local retention, sterility, injectability, aerosolization, or mucosal compatibility, depending on the route. Consequently, the key benefit of ASDs may lie in their integration into route-specific formulation architectures rather than replacing other solubilization methods. Nonetheless, such combinations can increase formulation complexity and regulatory challenges. Future research should assess whether hybrid ASD-based systems offer significant advantages over simpler options by examining biorelevant metrics like release, permeation, pharmacokinetics, pharmacodynamics, and safety questions.

## Future Directions: Toward Translationally Relevant ASD Design

To advance the field, future research should focus on translational relevance rather than small formulation tweaks. This involves creating biorelevant testing platforms that mimic route-specific physiological conditions, including limited fluid volumes, dynamic clearance, and barrier interactions. Additionally, increasing collaboration between *in vitro* and ex vivo models is essential for developing more reliable performance predictors prior to clinical trials [65, 66].

Although ASDs have been predominantly developed for poorly water-soluble small-molecule drugs, their underlying principles may also be relevant to larger molecular entities, including biologics, and other complex therapeutic molecules. In these cases, however, the formulation objective is not restricted to enhancing apparent solubility or generating supersaturation.

Larger molecules are often sensitive to conformational changes, aggregation, chemical degradation, interfacial stress, moisture, and processing conditions [113]. Therefore, applying ASD concepts to these molecules requires a broader interpretation of solid-state stabilization, in which the amorphous matrix may contribute not only to dissolution modulation but also to preserving molecular structure and biological activity during storage and administration. Thus, larger molecular entities represent an emerging frontier for ASD research, but their development will require dedicated characterization strategies and translational models that go beyond conventional small-molecule assessments of ASD.

Furthermore, computational tools and data-driven methods can aid in the more rational selection of drug–polymer systems and improve predictions of stability and release behavior. Nonetheless, these tools must be validated with experimental and clinical data to confirm their applicability in real-world settings [16].

Ultimately, the focus should shift from showing supersaturation to proving therapeutic benefit. Conducting comparative studies with current formulations, assessing clinically relevant endpoints, and developing route-specific optimization strategies will be crucial to translating theoretical potential into real clinical application. The effective translation of ASDs in non-oral drug delivery relies on both advancing formulation science and harmonizing mechanistic insights with physiological realities and clinical requirements.

## Conclusion

Amorphous solid dispersions (ASDs) are a flexible strategy in solid-state technology aimed at enhancing the effectiveness of poorly water-soluble drugs, extending their use beyond oral administration. In non-oral delivery methods, they help stabilize drugs in high-energy amorphous forms, improve apparent solubility, and facilitate transient supersaturation—supporting either local or systemic drug exposure. Nonetheless, these benefits are highly dependent on route-specific factors such as fluid availability, clearance rates, tissue barriers, residence time, local retention, and the properties of the final dosage form.

Current literature evidence shows that non-oral ASDs have evolved from initial proof-of-concept amorphization studies to more diverse route-specific systems. Transdermal systems are currently the most advanced non-oral options, while the formulations intended for the other routes remain mainly in formulation and preclinical stages.

A key takeaway from this review is that ASDs should not be regarded as universal solutions for solubility enhancement or as inherently better than other formulation methods. Their effectiveness depends on how well amorphization, drug–polymer interactions, molecular mobility, phase behavior, and supersaturation can be optimized for the specific route, therapeutic goal, and secondary pharmaceutical base. Future progress will depend on biorelevant *in vitro*, *ex vivo*, and *in vivo* models, improved *in vitro*–*in vivo* correlations, and route-specific PK/PD endpoints;

Overall, non-oral ASDs are versatile platforms that may serve as viable alternatives for drug solubility-enhancement or as complements to other solubility-enhancing methods. Their successful application depends on demonstrating that physicochemical benefits translate into meaningful therapeutic effects, safety, patient benefits, and durable pharmaceutical products suited to each administration route. Ultimately, the future of non-oral ASDs will depend less on demonstrating improved solubility and more on proving route-specific therapeutic benefit through clinically meaningful translational evidence.

## Data Availability

No new data were created or analyzed in this study. Data sharing is not applicable to this review article.
